# Ionic Liquids Facilitate the Dispersion of Branched Polyethylenimine Grafted ZIF-8 for Reinforced Epoxy Composites

**DOI:** 10.3390/polym15081837

**Published:** 2023-04-10

**Authors:** Junchi Ma, Shihao Zhou, Yuanchang Lai, Zhaodi Wang, Nannan Ni, Feng Dai, Yahong Xu, Xin Yang

**Affiliations:** Key Laboratory for Light-Weight Materials, Nanjing Tech University, Nanjing 210009, Chinaxin12.25@hotmail.com (X.Y.)

**Keywords:** BPEI-ZIF-8/IL, epoxy composite, mechanical properties, thermal properties, damping properties, dielectric properties

## Abstract

Metal–organic frameworks (MOFs) have been previously shown as an emerging modified class of epoxy resin. In this work, we report a simple strategy for preventing zeolitic imidazolate framework (ZIF-8) nanoparticles from agglomerating in epoxy resin (EP). Branched polyethylenimine grafted ZIF-8 in ionic liquid (BPEI-ZIF-8) nanofluid with good dispersion was prepared successfully using an ionic liquid as both the dispersant and curing agent. Results indicated that the thermogravimetric curve of the composite material had no noticeable change with increasing BPEI-ZIF-8/IL content. The glass transition temperature (T*_g_*) of the epoxy composite was reduced with the addition of BPEI-ZIF-8/IL. The addition of 2 wt% BPEI-ZIF-8/IL into EP effectively improved the flexural strength to about 21.7%, and the inclusion of 0.5 wt% of BPEI-ZIF-8/IL EP composites increased the impact strength by about 83% compared to pure EP. The effect of adding BPEI-ZIF-8/IL on the T*_g_* of epoxy resin was explored, and its toughening mechanism was analyzed in combination with SEM images showing fractures in the EP composites. Moreover, the damping and dielectric properties of the composites were improved by adding BPEI-ZIF-8/IL.

## 1. Introduction

Epoxy resin (EP) has been widely used in aerospace, microelectronics manufacturing, and automobile industries due to its corrosion, high thermal stability, and excellent mechanical properties [1,2,3,4]. However, the significant disadvantages such as high brittleness, poor toughness, and low flame retardancy have limited its utilization. Therefore, epoxy toughening has long been a topic of intense attention in various fields.

The toughness of EP can be achieved effectively by incorporating suitable second-phase materials such as rubber elastomers, thermoplastic resins, thermotropic liquid crystalline polymers, and inorganic nanoparticles. However, some critical problems are emerging for these second-phase materials used for toughening epoxy resin. The incorporation of rubber elastomers into EP is a feasible strategy to increase the toughness by forming an island structure with the matrix. When impacted, the stress concentration causes rubber particles to induce silver streaks, generate shear bands, and absorb a lot of energy [5]. In addition, rubber-toughened epoxy resins can lead to decreases in the strength and modulus of the material [6]. Moreover, thermotropic liquid crystalline polymers are difficult to source and complex to synthesize. They also have high melting points. In addition, they are less compatible with epoxy resins [7]. Although inorganic nanoparticle-based EP composites can largely overcome the brittleness and limited mechanical strength of polymers, nanoparticles in a resin matrix can cause agglomeration due to their high specific surface area and high surface activity, resulting in unsatisfactory results. One strategy to overcome the agglomeration phenomenon of nanoparticles is to modify the surface of nanoparticles [8]. A solvent-free nanofluid is an organic–inorganic material with grafted organic chains as the shell and nanoparticles as the core [9,10]. It can make nanoparticles behave like liquids in the absence of a solvent, thus successfully preventing nanoparticle aggregation. Solvent-free nanofluids including carbon nanotube solvent-free nanofluid [11] and solvent-free ionic silica nanofluid [12] have been widely used for more than a decade.

Metal–organic frameworks (MOFs) are porous crystalline materials formed by coordination reactions between metal ions and organic ligands. They are nanomaterials with a wide range of applications such as gas capture and separation [13,14]. They have advantages such as large specific surface area, easy functionalization, permanent pores, and high stability. In recent years, MOFs have also been applied to modify the resin matrix [15,16]. Some researchers have grafted MOF onto other materials and then mixed them with epoxy. For example, Ma et al. [17] synthesized polyaniline (PANI) modified Sn-MOF to enhance the fire resistance of EP. Other researchers have applied MOF material modification to EP. For example, Zhang et al. [18] reported that when UiO-66 is used to connect PBA and PDA as a fire retardant in EP, it can decrease the carbon monoxide and peak heat release rates. Zeolitic imidazolate framework (ZIF) material is a typical MOF. ZIFs have attracted increased attention because of their crystallinity, exceptional porosity, and rich structural diversity. ZIF-8 is the most investigated among the various ZIFs. Previous research has shown that ZIF-8 can be used for toughening resins due to its high specific surface area, low dielectric constant, and low polarity [19,20,21]. However, like other nanoparticles, ZIF-8 faces the challenge of being uniformly dispersed in the resin matrix for achieving an excellent modification due to the same problem of agglomeration. Shi et al. [22] blended ZIF-8 with PLA and found that both mechanical properties and flame retardancy of PLA/ZIF-8 nanocomposites are obviously improved compared with those of pure PLA. Zhao et al. [23] synthesized ZIF 8@modifed sepiolite hybrid with an in situ grown method and used it to modify epoxy resin. Their results indicated that when the addition amount was 0.6 wt%, the heat decomposition temperature was increased by 46.2 °C, and the T*_g_* was increased by 8.5 °C, whereas the dielectric constant of EP was reduced by 0.66. Nevertheless, the previously published studies were limited in addressing the agglomeration problem of ZIF-8 well or they failed to make a comprehensive study on epoxy resin.

Ionic liquids (ILs) are organic molten salts that usually appear as liquids at room temperature. ILs have a few advantages, including high solvent capacity [24,25,26], excellent chemical and thermal stability [27,28], low vapor pressure [29,30], high dielectric constant [31,32], and a wide electrochemical window [33,34]. At present, ILs have become a research hotspot in the fields of chemistry, physics, and engineering [28]. Recently, structurally related imidazolium-based ILs have also been used as curing or toughening agents in epoxy resins because imidazoles are a very important class of epoxy resin curing agents with long service life and low volatility [35,36,37,38]. Existing research studies on ILs have demonstrated that adding ionic liquids to epoxy resins can reduce the agglomeration of nanoparticles [39] while simultaneously enhancing the mechanical properties [40,41], flame-retarding ability [42], and frictional characteristics [43] of epoxy resins. In addition, Dai et al. [44] developed a third class of porous liquids by rationally coupling metal–organic framework nanoparticles with bulky ILs. In this study, long-chain ionic liquids are used to prevent the solvent molecules or ions from accessing the pore channels within the MOFs while maintaining a uniform and stable dispersion in the MOFs in solvents.

Inspired by previous reports, we proposed a new method to reduce the agglomeration of MOFs. We modified ZIF-8 and prepared branched polyethylenimine-grafted ZIF-8 (BPEI-ZIF-8) with an in situ synthesis method. The BPEI-ZIF-8 that was created using this technique contained a significant quantity of -NH_2_ on the surface, which could chemically react with epoxy groups. BPEI-ZIF-8 and ionic liquids were combined to obtain BPEI-ZIF-8/IL to ensure that BPEI-ZIF-8 could maintain good dispersion. When BPEI-ZIF-8/IL was added into epoxy resin, the BPEI-ZIF-8 and ILs could be chemically linked with the resin matrix. Imidazolium-base ILs could also promote the curing of epoxy resin. The mechanical, thermal, damping, and dielectric properties of the cured BPEI-ZIF-8/IL/EP composites with increasing BPEI-ZIF-8/IL nanofluid content were then systematically investigated.

## 2. Materials and Methods

### 2.1. Materials

4,4’-Diaminodiphenylsulfone (DDS, Yinsheng Chemical Co., Ltd., Suzhou, China). Diglycidyl ether bisphenol-A (DGEBA) epoxy resin (E-54, Xingchen Synthetic Material Co., Ltd., Nantong, China). Epoxy resin (AG-80, Huayi Resin Co., Ltd., Shanghai, China). Zn(NO_3_)_2_·6H_2_O (99.99% metals basis), branched polyethyleneimine (BPEI, average M.W. 600, 99%), and 2-methylimidazole (Hmim, 98%, Aladdin Reagent Co., Ltd., Shanghai, China). Methanol (CH_3_OH, Lingfeng Chemical Reagent Co., Ltd., Shanghai, China). 1-Butyl-3-heptylimidazolium bromide (Northwestern Polytechnical University, Xi’an, China). None of these chemicals was further purified before usage.

### 2.2. Preparation of ZIF-8

ZIF-8 was prepared according to a previous report [45]. Briefly, Zn(NO_3_)_2_·6H_2_O (1.472 g, 5 mmol) and Hmim (3.248 g,40 mmol) were dissolved in 80 mL and 120 mL of methanol, respectively. The Zn(NO_3_)_2_·6H_2_O solution was then added to the Hmim solution and continuously stirred at room temperature for 60 min. The suspension was centrifuged at 8000 RPM for 15 min and then washed repeatedly with methanol. The product was then put into a vacuum oven to dry at 80 °C for 12 h.

### 2.3. Preparation of BPEI-ZIF-8

BPEI-ZIF-8 was prepared following a published method [45,46]. Briefly, BPEI (0.6 g, 1 mmol) was added into the Hmim solution before mixing with the Zn(NO_3_)_2_·6H_2_O solution. The mixture was continuously stirred at 25 °C for 12 h. The mixed solution was then centrifuged at 10,000 RPM for 10 min and washed with methanol three times. The product was then dried in a vacuum oven for 12 h at 80 °C to obtain BPEI-ZIF-8 powder.

### 2.4. Preparation of BPEI-ZIF-8/IL

BPEI-ZIF-8 (1.87 g) was added into a long-chain imidazole-type ionic liquid (18.34 g, 82.1 mmol) containing methanol. The final product was dried under vacuum at 80 °C for 12 h to remove methanol and water.

### 2.5. Preparation of BPEI-ZIF-8/IL/EP Composites

**Scheme 1:**
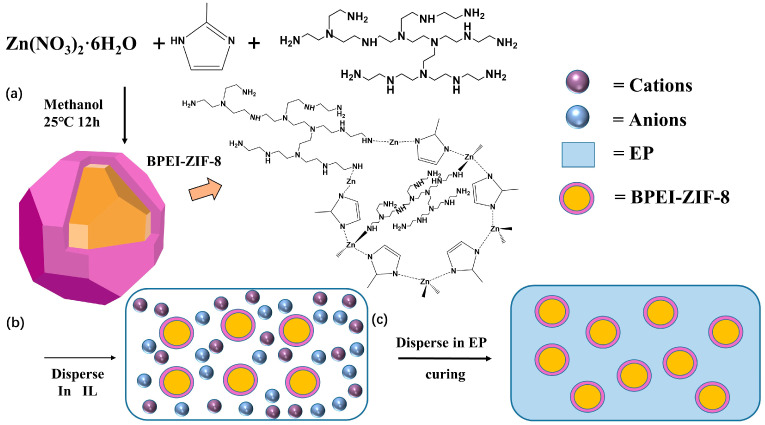
(**a**) Synthesis of BPEI-ZIF-8 nanoparticles. (**b**) Synthesis of BPEI-ZIF-8/IL. (**c**) Preparation of BPEI-ZIF-8/IL/EP composites.

First, 40 phr of AG-80 and 40 phr of E-54 were mixed. After a high-speed stirring at 90 °C for 20 min, different proportions (0 wt%, 0.2 wt%, 0.5 wt%, 1 wt%, 2 wt%) of BPEI-ZIF-8/IL were added, and the mixture was ultrasonically mixed for one hour and then stirred at 1500 r/min for 1 h. After adding 30 phr of DDS, the mixture was heated up to 120 °C and stirred at a high speed of 1800 r/min for 30 min until the DDS was completely dissolved in the resin. After vacuuming at 110 °C for half an hour, the product was poured into a polytetrafluoroethylene mold and cured at 130 °C for 1 h, 160 °C for 1 h, 180 °C for 2 h, and 200 °C for 2 h. Scheme 1 and Table 1 show the specific experimental steps and experimental formulations of the products.

### 2.6. Characterization

Fourier transform infrared spectroscopy (FTIR) was used to study the chemical structures of ZIF-8 and BPEI-ZIF-8. FTIR spectra were recorded using a Bruker’s Tensor II spectrometer in the range of 500 to 4000 cm^−1^ at a resolution of 4 cm^−1^. An X-ray diffractometer (XRD) analysis was carried out using a Smartlab SE between 5° and 55° under a 9 kW power.

Thermogravimetric analysis (TGA) was performed with a TGA550 TA instrument under nitrogen ambiance with a heating rate of 10 °C/min from 30 °C to 700 °C. Differential scanning calorimetry (DSC) analysis was performed using a DSC250 TA instrument in a nitrogen atmosphere with a heating rate of 10 °C/min and a temperature range of 30–300 °C. Before testing, all samples were dried at 80 °C for 12 h.

The flexural strength test was carried out for nanocomposites with an electronic universal testing machine (Xinsansi Material Testing Company, Shenzhen, China). The size of each sample was (80 ± 0.02) mm × (15 ± 0.02) mm × (4 ± 0.02) mm according to previous reports [11].For the impact test, the sample was subjected to an impact power load at a speed of 2.9 m/s using a PTM-7000 impact testing machine (SUNS Co., Ltd., Shenzhen, China). The size of the sample was (80 ± 0.02) mm × (10 ± 0.02) mm × (4 ± 0.02) mm according to previous reports [11].

Surface and fracture morphologies of pure EP and EP composites were investigated with a scanning electron microscope (SEM, TESCAN S8000). All samples were gold-plated prior to testing.

The dielectric properties of epoxy resins were tested with a Broadband Dielectric spectrometer (Concept 80, Novocontrol, Germany) at room temperature. The dimension of each sample was (10 ± 0.02) mm × (10 ± 0.02) mm × (3 ± 0.02) mm, and the test range was 10–10^6^ Hz. A silver paste was used to coat the top and bottom sides of the samples.

Dynamic mechanical analysis (DMA, Q800, TA, USA) was performed under a N_2_ atmosphere with a heating rate of 3 °C/min from 30 °C to 300 °C in a single cantilever mode and a frequency of 1 Hz. The sample dimension was (40 ± 0.02) mm × (10 ± 0.02) mm × (3 ± 0.02) mm.

## 3. Results and Discussion

### 3.1. Characterization of BPEI-ZIF-8/IL Nanoparticles

**Figure 1:**
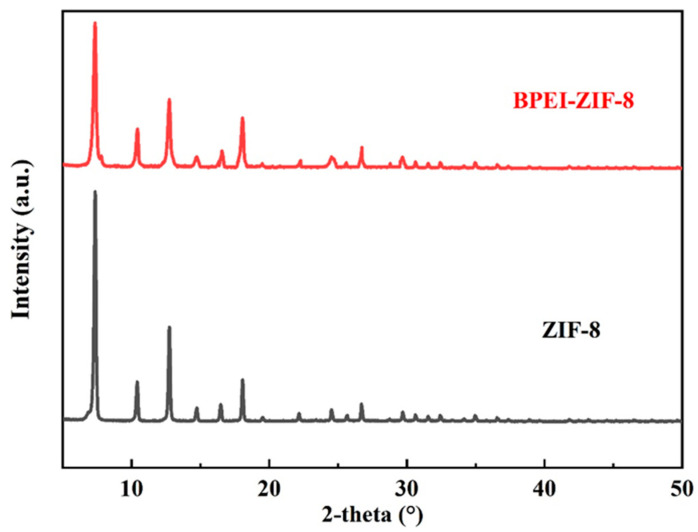
XRD spectra of ZIF-8 and BPEI-ZIF-8.

The crystal structures of ZIF-8 and BPEI-ZIF-8 were characterized using XRD (Figure 1). Both ZIF-8 and BPEI-ZIF-8 showed three major peaks at 2-theta values of 7.35°, 10.43°, and 12.75° indexed to the (011), (002), and (112) crystal planes, respectively, without obvious miscellaneous peaks. The peak intensity of BPEI-ZIF-8 was lower than that of ZIF-8, which might be due to the decrease in ZIF-8 content and the enhancement in the interaction between the PEI and Zn^2+^ sites [46,47,48]. These results were consistent with previous reports [20,46], demonstrating successful syntheses of ZIF-8 and BPEI-ZIF-8.

**Figure 2:**
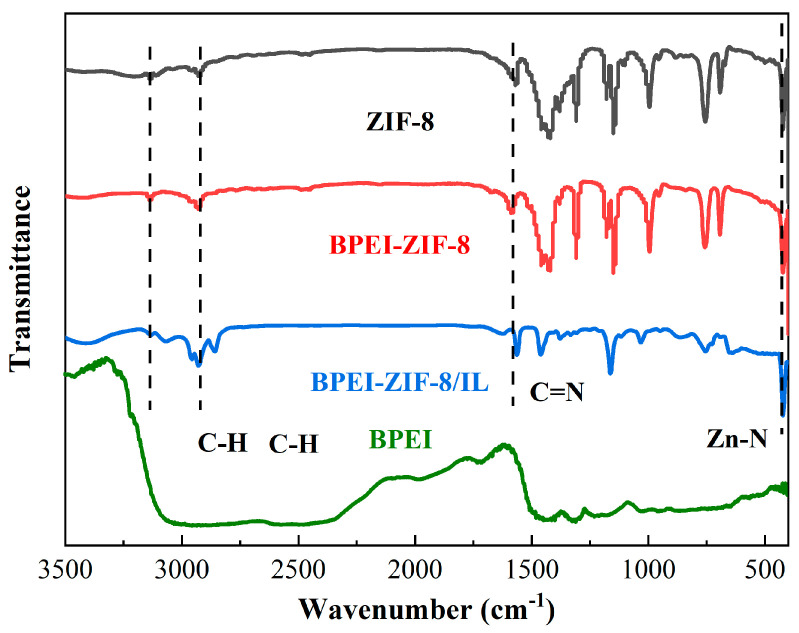
FTIR spectra of ZIF-8, BPEI, BPEI-ZIF-8, and BPEI-ZIF-8/IL.

Figure 2 illustrates the spectra of ZIF-8, BPEI, BPEI-ZIF-8, and BPEI-ZIF-8/IL. The stretching vibration peak in Zn-N appeared at 425 cm^−1^. The stretching mode in 2-methylimidazole C=N appeared at 1570 cm^−1^. The stretching vibration peaks in the aromatic ring in 2-methylimidazole and the C-H graft side chain on BPEI-ZIF-8 appeared at 2930 cm^−1^ and 3135 cm^−1^, respectively. Figure S1 illustrates the FTIR spectra of BPEI and BPEI-ZIF-8. The stretching peak in C-N appeared at 1043 cm^−1^ and 1127 cm^−1^, respectively. The N-H bending peaks for NH_2_ and NH vibration appeared at 1361 cm^−1^ and 1452 cm^−1^, respectively [49,50]. Most of the characteristic peaks of ZIF-8 also appeared in the FTIR spectrum of BPEI-ZIF-8/IL. Moreover, the characteristic peak at 2930 cm^−1^ of BPEI-ZIF-8/IL was more obvious because of the -CH peak on IL. Therefore, the successful preparation of BPEI-ZIF-8/IL was confirmed with the FTIR analysis.

### 3.2. Thermal Behavior

**Figure 3:**
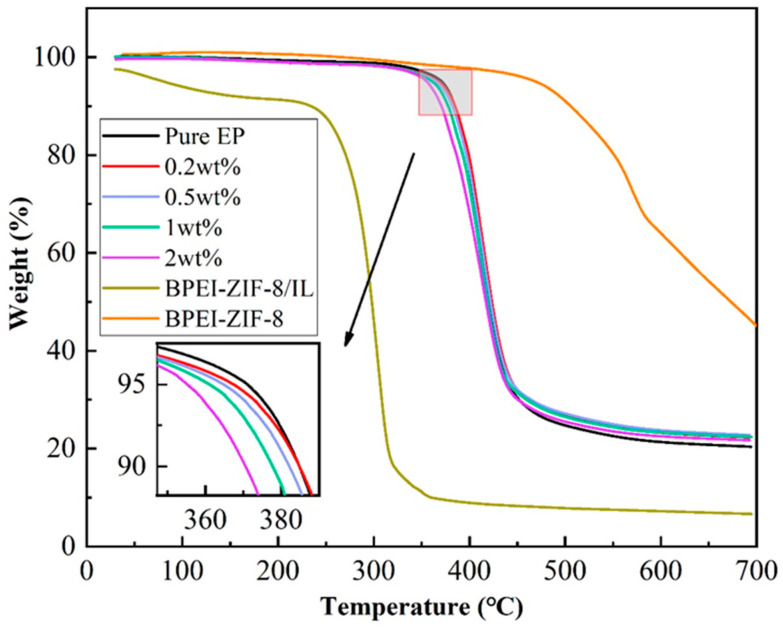
TGA curves of pure EP and BPEI-ZIF-8/IL/EP composites with different contents of BPEI-ZIF-8/IL.

The thermal stabilities of the epoxy composites were characterized using TGA analysis. As shown in Figure 3, the thermogravimetric curves of all epoxy resin composites were basically the same, showing no significant mass change in composite until 300 °C with an obvious weight loss starting at about 350–500 °C. Furthermore, the weight loss rate of the BPEI-ZIF-8/IL/EP composite was slightly higher than that of pure EP. This might be attributed to the thermal stability of BPEI-ZIF-8/IL. BPEI-ZIF-8/IL was decomposed in two parts, the first weight loss at 50 °C to 210 °C and the second weight loss between 250 °C and 350 °C. The first weight loss in BPEI-ZIF-8/IL is possibly due to evaporation, and the second weight loss was observed at around 250 °C, owing to the decomposition of ionic liquids. The results are similar to previous studies on other ionic liquids [38,51]. The mass of BPEI-ZIF-8/IL did not change much after 400 °C. Figure 3 also shows that the addition of BPEI-ZIF-8 did not change the main structure of the EP resin, leading to excellent thermal stability. The residual carbon amounts of all the epoxy composite samples were higher than that of pure EP when the samples reached 700 °C because BPEI-ZIF-8/IL nanofluid still had some residual carbon at 700 °C. It also can be seen from Table S1 that BPEI-ZIF-8/IL/EP composites with different contents of BPEI-ZIF-8/IL represented similar thermal stability.

**Figure 4:**
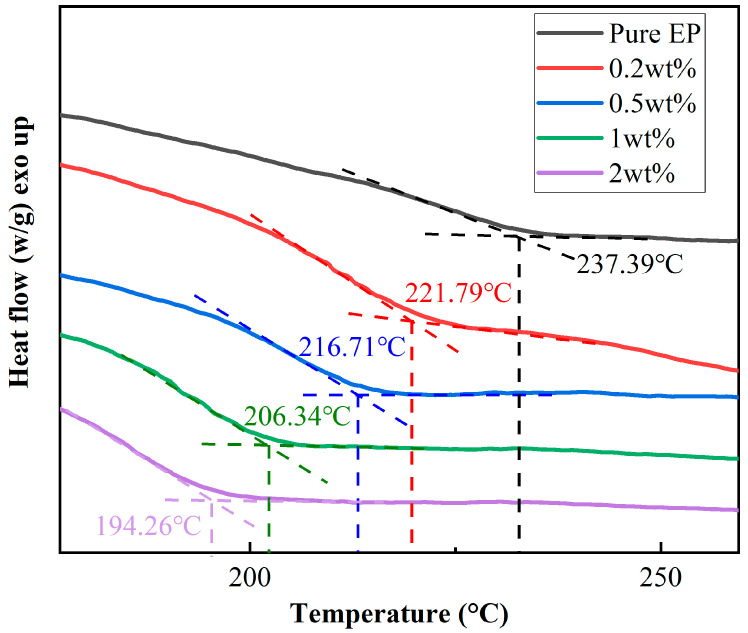
DSC curves of pure EP and BPEI-ZIF-8/IL/EP composites with different contents of BPEI-ZIF-8/IL.

Figure 4 shows the DSC curves of the EP and BPEI-ZIF-8/IL nanofluid/epoxy resin composites. It can be seen that the Tg of pure EP was the highest. With increasing content of the BPEI-ZIF-8/IL nanofluid, the Tg of EP composite presented a decreasing trend. With the addition of 2 wt% BPEI-ZIF-8/IL nanofluid, Tg decreased from 237.39 °C to 194.26 °C, representing a decrease of 18.17%. This result might be because branched polyethyleneimine grafted on ZIF-8 reacted with the epoxy group to form a branched structure, leading to a decrease in T*_g_* of the EP. It might also be because the long-chain, imidazole-type ionic liquid accelerated the movement in the molecular chain, and the long-chain imidazole-type ionic liquid can also act as a curing agent for epoxy resins, making the long chains of EP cross-linked shorter.

### 3.3. Mechanical Properties of BPEI-ZIF-8/IL/EP Composites

**Figure 5:**
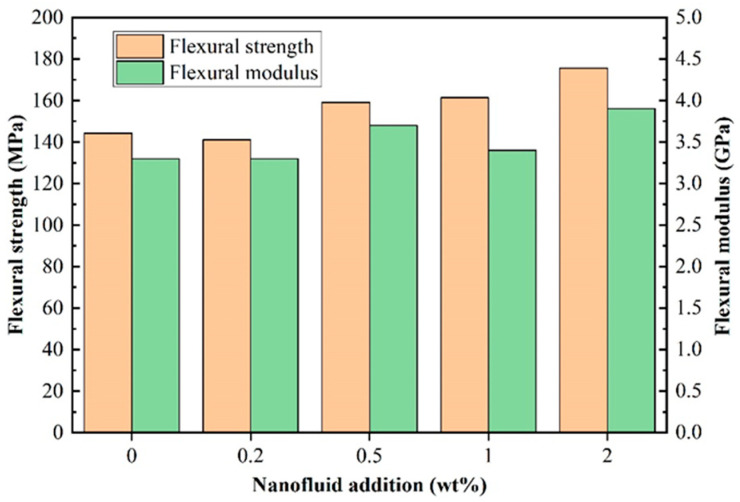
Flexural strength and flexural modulus of BPEI-ZIF-8/IL/EP composites.

The effects of adding different levels (0, 0.2 wt%, 0.5 wt%, 1 wt%, 2 wt%) of BPEI-ZIF-8/IL on the mechanical properties of BPEI-ZIF-8/IL/EP composites were examined by performing bending tests and impact tests. As shown in Figure 5, both the flexural strength and flexural modulus were increased when the level of BPEI-ZIF-8/IL nanofluid added was increased from 0 to 2 wt%. When the content of BPEI-ZIF-8/IL nanofluid was 2 wt%, the flexural strength and flexural modulus peaked at 175.6 Mpa and 3.9 Gpa, respectively, which were 21.7% and 15.6% higher than those of pure EP (144 MPa and 3.34 GPa), respectively. The flexural strength and flexural modulus of 0.2 wt% EP composites were decreased slightly compared with that of pure EP, possibly due to errors during testing.

**Figure 6:**
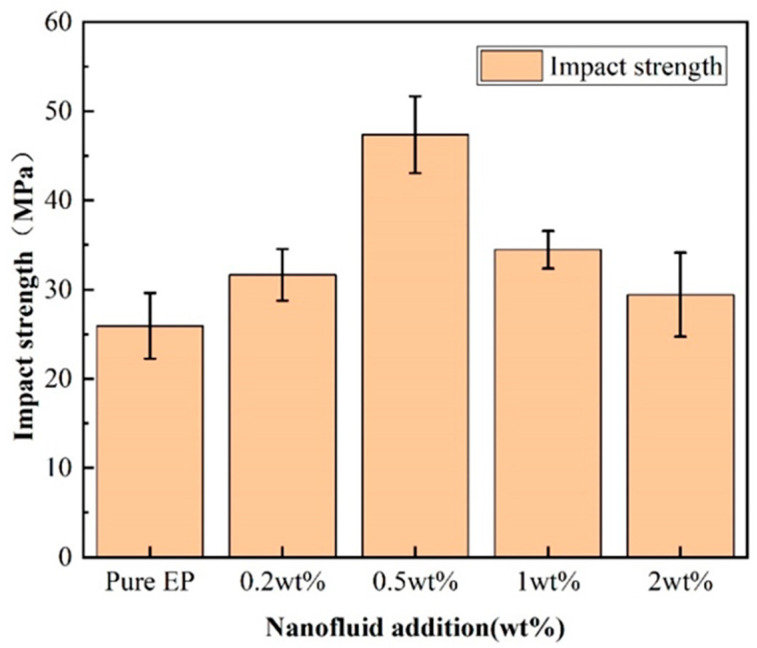
Impact strength of BPEI-ZIF-8/IL/EP composites.

Figure 6 shows the impact strength of BPEI-ZIF-8/IL/EP composites. The impact strength tended to increase initially. It was then decreased when the amount of BPEI-ZIF-8/IL was increased from 0 to 2 wt%, as can be seen in Figure 4. By contrast, 0.5 wt% BPEI-ZIF-8/IL led to an 83% increase in impact strength from 25.9 to 47.4 kJ/m^2^, which was the peak value. When the level of BPEI-ZIF-8/IL nanofluid was further increased to 1 wt% and 2 wt%, both impact strengths were lower than that of the BPEI-ZIF-8/IL/EP composite with BPEI-ZIF-8/IL content at 0.5 wt% [52]. However, the impact strengths of all composites were higher than the impact strength of pure EP.

### 3.4. Toughening Mechanism

**Figure 7:**
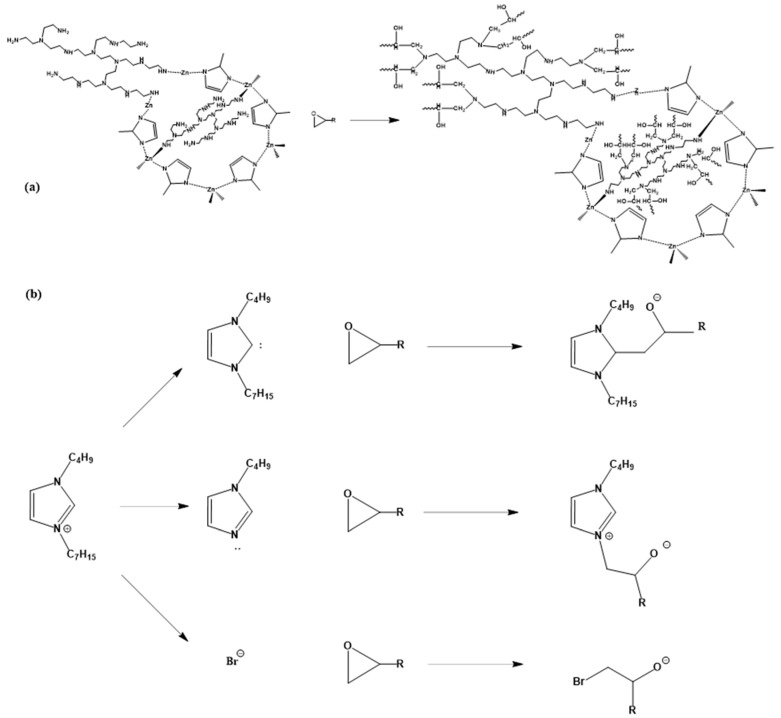
Chemical reactions between BPEI-ZIF-8/IL and EP (**a**) reaction of BPEI-ZIF-8 and epoxy ring, (**b**) reactions of ionic liquid and epoxy ring.

The reactions after adding BPEI-ZIF-8/IL nanofluid to epoxy resin are shown in Figure 7. When BPEI-ZIF-8/IL nanofluid was used to toughen epoxy resins, the ionic liquid not only dispersed BPEI-ZIF-8 but also sped up the crosslinking and curing of the EP. The ionic liquid could react with EP through three reactions: a carbene reaction, an imidazole reaction, and a nucleophilic reaction [38]. An ionic liquid can react with epoxy resin in which imidazole-type ionic liquid deprotonation to form a carbanion structure starts at about 60 °C. In the imidazole reaction, 1-butyl 3 heptyl imidazole dealkylation starts at about 90 °C. The nucleophilic reaction requires a higher temperature and a wider range of reaction temperatures than previous reactions.

On the other hand, the –NH_2_ on the surface of the BPEI-ZIF-8 particles can better react with the epoxy groups and hydroxyl groups of the EP to form chemical bonds (Figure 7a). These chemical bonds can enhance the interfacial compatibility of the composite and improve the crosslinking density of the polymer matrix. However, according to the DSC curves of the EP composites (Figure 4), the Tg values of the EP composites decreased gradually with the addition of fluid because of BPEI-ZIF-8/IL branched by the reaction with epoxy groups. These results suggest that the reduction effect of the branched structure on EP is greater than the improvement effect of the chemical bond produced by the reaction of the amino group of BPEI-ZIF-8/IL and epoxy group on the EP composites. Finally, the BPEI-ZIF-8 grafted BPEI chain could serve as a bridge between ZIF-8 and epoxy resin. As a result, when the material is subjected to external energy, it may more effectively transmit the energy to nanoparticles, thus improving the toughening result [53].

**Figure 8:**
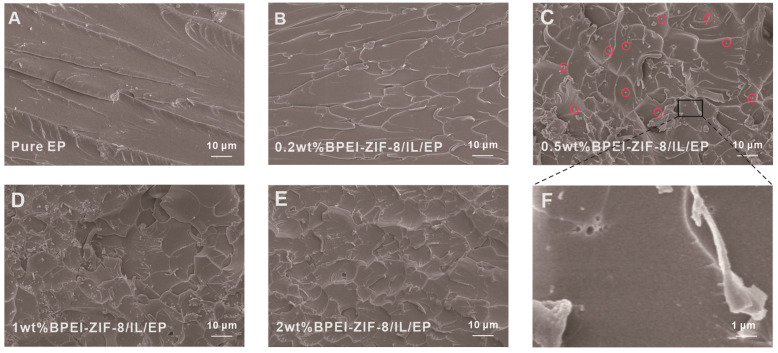
SEM images of BPEI-ZIF-8/IL/EP composites with fractures (**A**) Pure EP, (**B**) 0.2 wt% BPEI-ZIF-8/IL/EP, (**C**) 0.5 wt% BPEI-ZIF-8/IL/EP, (**D**) 1 wt% BPEI-ZIF-8/IL/EP, (**E**) 2 wt% BPEI-ZIF-8/IL/EP, (**F**) A partial enlargement of the 0.5 wt% BPEI-ZIF-8/IL/EP.

The main mechanisms involved in the toughening of epoxy resin by nanoparticles are cavity growth, crack deflexion, crack bridging, crack branching, and so on [54]. To better illustrate that the addition of BPEI-ZIF-8/IL could have a toughening effect on the epoxy resin, SEM was used to observe the fracture morphologies of the epoxy resin composites (Figure 8). It can be seen from Figure 8A that the EP fracture was relatively smooth, and the crack extension direction was relatively consistent, indicating a typical brittle fracture. The fracture morphologies of the epoxy composites are shown in Figure 8B–E. It was found that the fracture crack trend became more complex, with crack deflection, branching, and cavity gradually increasing, all of which could cause the resin matrix to absorb more energy. These results also confirmed that the fractures in the EP composites showed ductile fractures. Moreover, uniformly dispersed BPEI-ZIF-8 particles can be seen in Figure 8C, indicating that it is feasible to use IL to prevent the aggregation of BPEI-ZIF-8. Compared to the other EP composites, 0.5 wt% BPEI-ZIF-8/IL/EP exhibited rougher fracture, consistent with the result that the impact strength of 0.5 wt% BPEI-ZIF-8/IL/EP was the highest, as shown in Figure 8.

### 3.5. Damping Properties and Dynamic Mechanical Properties

**Figure 9:**
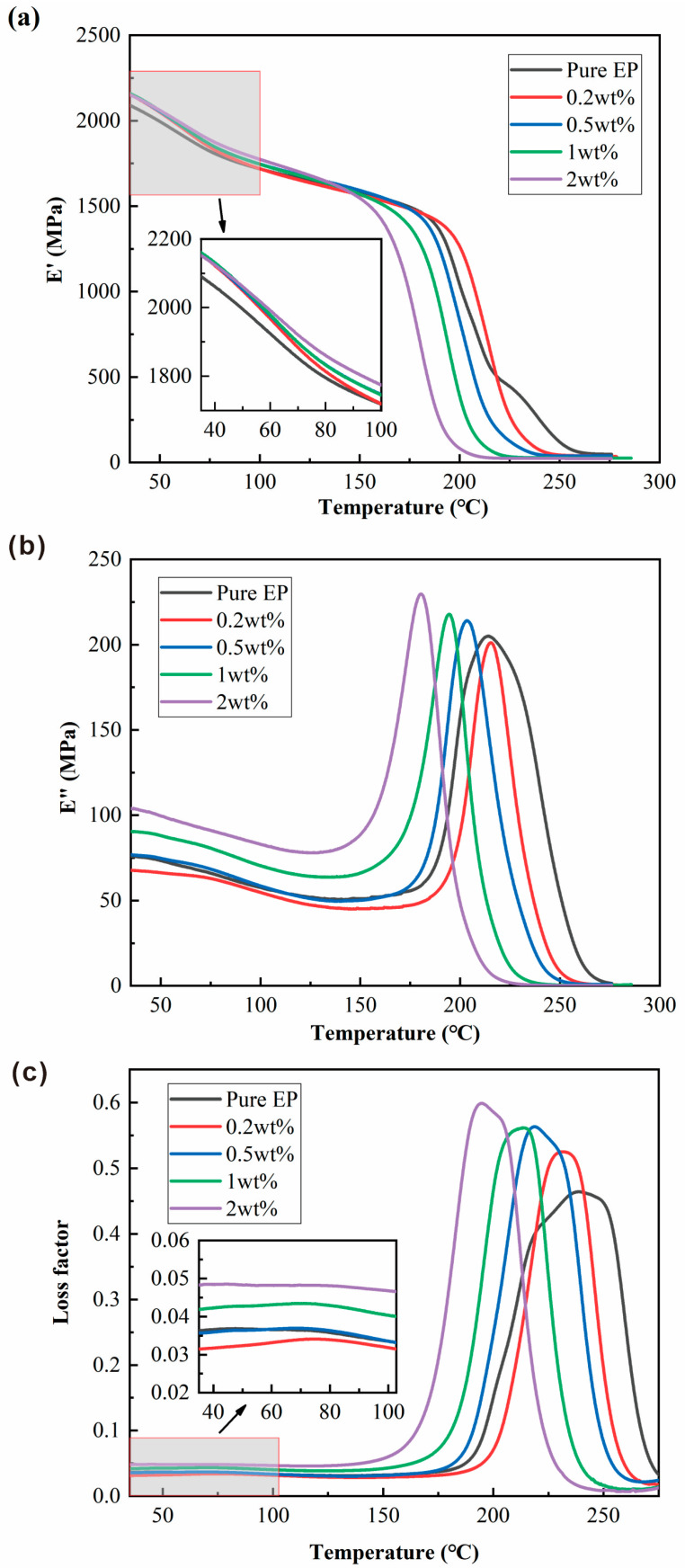
(**a**) Storage modulus, (**b**) loss modulus, and (**c**) loss factor of the BPEI-ZIF-8/IL/EP composites with different addition levels (wt%) of nanofluid.

The effects of different addition levels of BPEI-ZIF-8/IL nanofluid on the damping properties of the EP composites were examined using DMA. The results are shown in Figure 9. Figure 9a shows that the initial storage modulus (E’) values of the EP composites are greater than those of pure EP. Previous studies have tested the DMA of Epoxy@GMA-UiO-66 and Epoxy@NH_2_-UiO-66 and showed that their storage modulus is lower than that of pure EP [55]. With an increasing temperature, the storage modulus retention value of the 0.2 wt% EP composite was slightly higher than that of pure EP, whereas storage modulus retention values of the 0.5 wt%, 1 wt%, and 2 wt% EP composites were lower than that of pure EP. This result might be due to the interactions of BPEI-ZIF-8 and ILs with the epoxy remaining strong at lower temperatures. However, as the temperature increased, the epoxy would transit from a glassy state to a rubbery state, making it easier to travel between chain segments, thus reducing the constraint of BPEI-ZIF-8 and ILs on EP. As mentioned in the previous section on heat resistance, at high temperatures, long-chain imidazole-type ILs and branching produced by BPEI-ZIF-8 in EP could lead to easier movement between chain segments of EP composites, which are more likely to slip [11,56].

Figure 9b illustrates the loss modulus values of pure EP and EP composites. The loss modulus values of the 0.5 wt%, 1 wt%, and 2 wt% EP composites were higher than the loss modulus of pure EP, indicating that after BPEI-ZIF-8/IL nanofluid addition, the friction between BPEI-ZIF-8/IL nanofluid and the epoxy matrix could dissipate energy. The damping property of an EP composite is usually measured using the loss modulus and loss factor [57]. The loss factor is the ratio of the loss modulus to the storage modulus. Figure 9c shows the loss factor results for nanocomposites with different addition levels of the filler. The loss factor values of all EP composites were higher than that of pure EP. A forward movement in the peak of the loss factor indicates a lower glass transition temperature, consistent with the Tg results determined using DSC (Figure 4). Moreover, the loss factor curves of all composites had a single peak, suggesting that BPEI-ZIF-8/IL nanofluid had good interfacial compatibility with epoxy resin, which proved the toughening mechanism described in the previous section.

### 3.6. Dielectric Property

**Figure 10:**
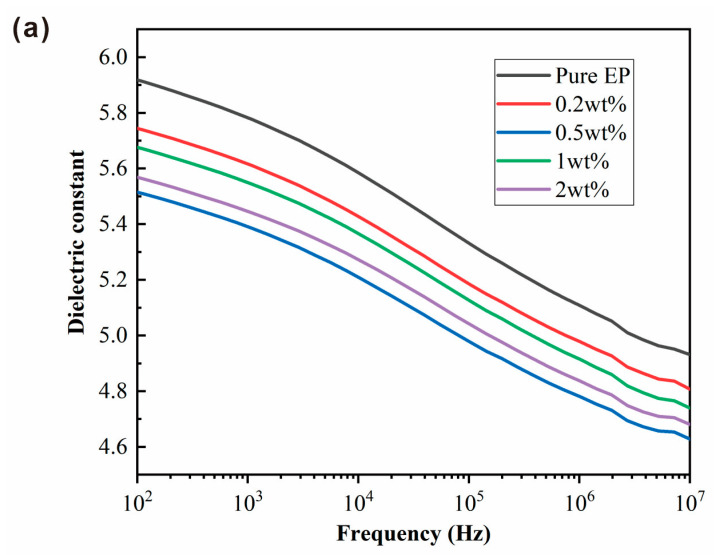

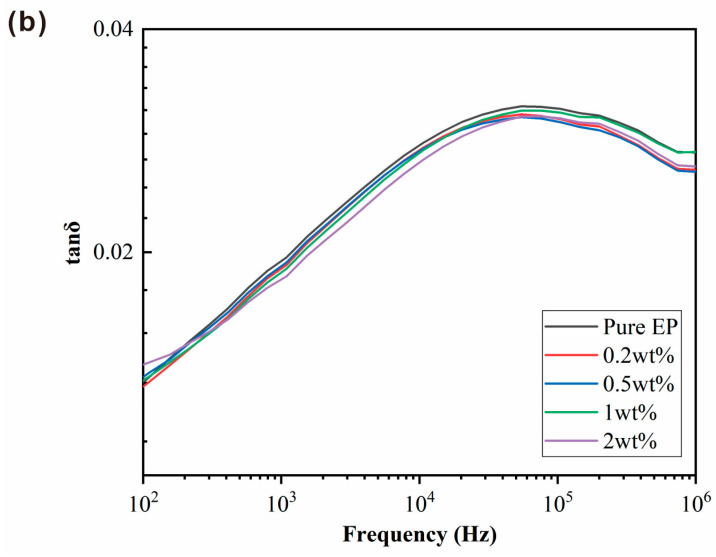
Dielectric constant (**a**) and dielectric loss (**b**) in BPEI-ZIF-8/IL/EP with different addition levels (wt%) of nanofluid.

The magnitude of the dielectric constant can be used to determine the degree of polarization in a material. Figure 10a,b shows dielectric constants and dielectric loss (tanδ) of BPEI-ZIF-8/IL/EP composites at room temperature with different frequencies. The dielectric constants of all BPEI-ZIF-8/IL/EP composites were lower than that of pure EP at the same frequency. They decreased with increasing frequency. Furthermore, among these composites, the 0.5 wt% nanofluid level resulted in the lowest dielectric constant. These results might be due to the presence of a large number of pores in BPEI-ZIF-8/IL that could reduce the dielectric constant [58]. The dielectric constant of an EP composite can be affected by electronic, ionic, dipolar, and surface polarization at a low frequency [59]. However, only electronic polarization plays a significant role at a high frequency. In addition, the amino group on BPEI-ZIF-8 could react with the epoxy group. Thus, the interfacial bonding between BPEI-ZIF-8 nanoparticles and epoxy resin is increased. The tight bonding of the two could inhibit the degree of polarization in EP [15]. The dielectric losses (Figure 10b) in all BPEI-ZIF-8/IL/EP composites were affected by the dielectric constant. They were all lower than the dielectric loss in pure EP.

## 4. Conclusions

In conclusion, a novel BPEI-ZIF-8/IL nanofluid with good dispersion was prepared as a toughening material. It was then incorporated into the EP matrix. The properties of the resulting epoxy composites were then investigated comprehensively. FTIR and XRD analyses demonstrated successful syntheses of BPEI-ZIF-8 nanoparticles and BPEI-ZIF-8/IL nanofluid. The glass transition temperature of the BPEI-ZIF-8/IL/EP composites decreased with increasing the addition level of the BPEI-ZIF-8/IL nanofluid. The addition of BPEI-ZIF-8/IL nanofluid did not change the excellent thermal stability of EP resin. The BPEI-ZIF-8/IL/EP composites showed improved flexural properties and impact strength. The mechanism involved in the improvement in the mechanical properties of EP composites by adding BPEI-ZIF-8/IL was explained, resulting in a change from brittle fractures for EP to ductile fractures for EP composites with SEM images. Moreover, the damping property of BPEI-ZIF-8/IL/EP composites was improved compared with that of pure EP. In addition, there were many pores on the BPEI-ZIF-8/IL material, which improved the dielectric properties of EP composites. This work provides a novel approach for preventing MOFs agglomeration and modifying epoxy resin with MOFs.

## Figures and Tables

**Table 1:** Formulations of EP composites with BPEI-ZIF-8/IL.

Sample	E-54 (phr)	Ag-80 (phr)	DDS (phr)	BPEI-ZIF-8/IL(wt%)
Pure EP	40	40	30	0
0.2 wt% BPEI-ZIF-8/IL/EP	40	40	30	0.2
0.5 wt% BPEI-ZIF-8/IL/EP	40	40	30	0.5
1 wt% BPEI-ZIF-8/IL/EP	40	40	30	1
2 wt% BPEI-ZIF-8/IL/EP	40	40	30	2

## Supplementary Materials

The following supporting information can be downloaded at: https://www.mdpi.com/article/10.3390/polym15081837/s1, Figure S1: FTIR spectra of BPEI, BPEI-ZIF-8; Table S1: TGA data of pure EP and BPEI-ZIF-8/IL/EP composites with different contents of BPEI-ZIF-8/IL; Video S1: BPEI-ZIF-8/IL.
