# Supplementary material for: Ionic Liquids Facilitate the Dispersion of Branched Polyethylenimine Grafted ZIF-8 for Reinforced Epoxy Composites
Source: Polymers (Basel). 2023 Apr 10;15(8):1837. doi: 10.3390/polym15081837 (PMC10146677; doi:10.3390/polym15081837)
Supplement: Supplementary file 1 [file polymers-15-01837-s001.zip › supplementary data.pdf]

Supplementary Information

Ionic liquids facilitate dispersion of branched polyethylenimine grafted ZIF-8 for reinforced epoxy composites

Junchi Ma, Shihao Zhou, Yuanchang Lai, Zhaodi Wang, Nannan Ni, Feng Dai * , Yahong Xu *, Xin Yang

Key Laboratory for Light-weight Materials, Nanjing Tech University, Nanjing 210009, China.

***E-mail: 201910006627@njtech.edu.cn (F. Dai), 201910006672@njtech.edu.cn (Y.H. Xu)**

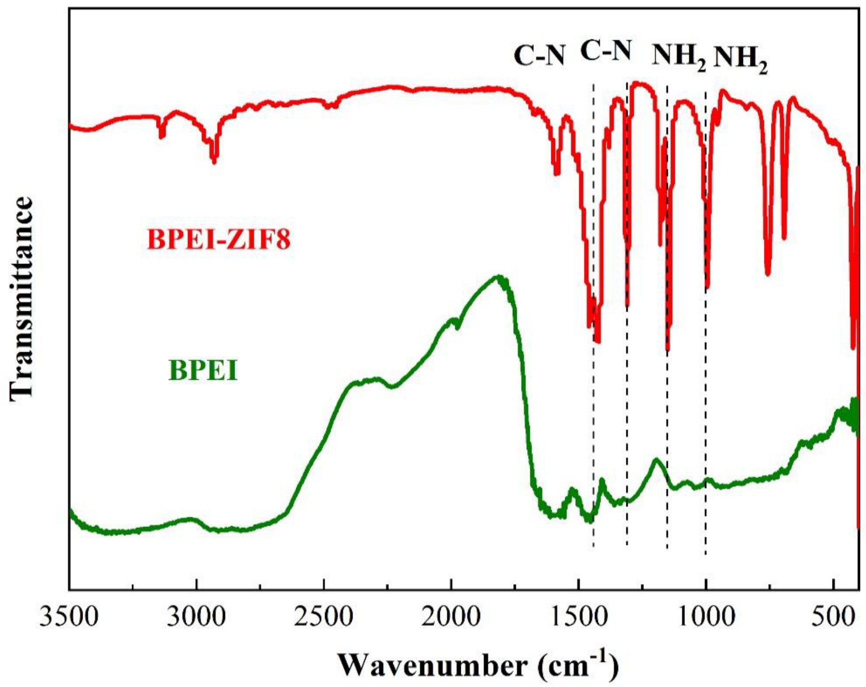

Figure S1. FTIR spectra of BPEI, BPEI-ZIF-8.

Figure S1 illustrates the FTIR spectra of BPEI and BPEI-ZIF-8. The stretching peak in C-N appeared at 1043 cm^{-1} and 1127 cm^{-1}, respectively. The N-H bending peaks for NH_2 and NH vibration appeared at 1361 cm^{-1} and 1452 cm^{-1}, respectively [49,50].

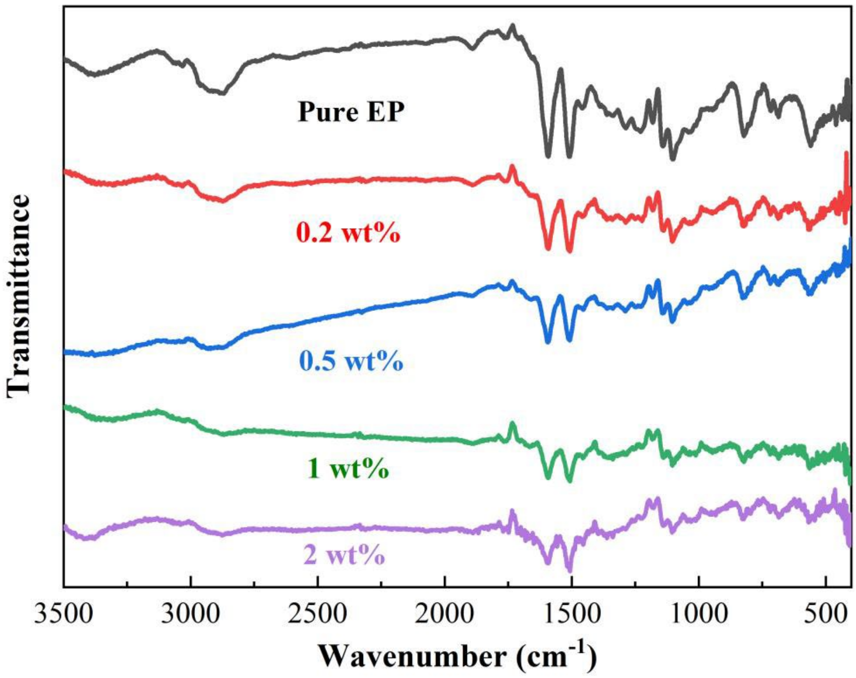

Figure S2. FTIR spectra of pure EP and BPEI-ZIF-8/IL/EP composites with different contents of BPEI-ZIF-8/IL.

As shown in Figure S2, spectra of all epoxy resin composites were roughly the same, the stretching mode of C=N appeared at 1570 cm^{-1}, the stretching vibration of -OH appeared at 3340 cm^{-1}. The stretching peak of C-N appeared at 1098 cm^{-1}, N-H bending peaks

for NH vibration at 1452 cm^{-1}, these results were consistent with previous reports [1-2]. The stretching vibration peak of Zn-N appeared at 425 cm^{-1}. Therefore, successful preparation of BPEI-ZIF-8/IL/EP were confirmed by FTIR analysis.

Table. S1. TGA data of pure EP and BPEI-ZIF-8/IL/EP composites with different contents of BPEI-ZIF-8/IL.

Sample	$T_{5\%}$ (°C)	$T_{10\%}$ (°C)	$T_{15\%}$ (°C)
Pure EP	371.62	385.30	392.11
0.2 wt% BPEI-ZIF-8/IL/EP	367.49	384.81	392.64
0.5 wt% BPEI-ZIF-8/IL/EP	364.85	382.15	390.19
1 wt% BPEI-ZIF-8/IL/EP	361.08	377.75	385.69
2 wt% BPEI-ZIF-8/IL/EP	355.30	370.60	378.83

It also can be seen from Table S1 that BPEI-ZIF-8/IL/EP composites with different contents of BPEI-ZIF-8/IL represented similar thermal stability.

References:

[1] D. Duraibabu, M. Alagar, S.A. Kumar, Studies on mechanical, thermal and dynamic mechanical properties of functionalized nanoalumina reinforced sulphone ether linked tetraglycidyl epoxy nanocomposites, Rsc Advances, 4 (2014) 40132-40140.

[2] K. Kanimozhi, K. Sethuraman, V. Selvaraj, M. Alagar, Development of ricehusk ash reinforced bismaleimide toughened epoxy nanocomposites, Frontiers in chemistry, 2 (2014) 65.
